# Identification of *Ganoderma* Disease Resistance Loci Using Natural Field Infection of an Oil Palm Multiparental Population

**DOI:** 10.1534/g3.117.041764

**Published:** 2017-06-05

**Authors:** Sébastien Tisné, Virginie Pomiès, Virginie Riou, Indra Syahputra, Benoît Cochard, Marie Denis

**Affiliations:** * Genetic Improvement and Adaptation of Mediterranean and Tropical Plant Research Unit (CIRAD, UMR AGAP), Montpellier, 34398 France; † P.T Socfindo Medan, 20001, Indonesia; ‡ PalmElit SAS, 34980 Montferrier sur Lez, France

**Keywords:** plant disease, multiparental population, quantitative disease resistance loci, survival analysis, identity-by-descent, MPP

## Abstract

Multi-parental populations are promising tools for identifying quantitative disease resistance loci. Stem rot caused by *Ganoderma boninense* is a major threat to palm oil production, with yield losses of up to 80% prompting premature replantation of palms. There is evidence of genetic resistance sources, but the genetic architecture of *Ganoderma* resistance has not yet been investigated. This study aimed to identify *Ganoderma* resistance loci using an oil palm multi-parental population derived from nine major founders of ongoing breeding programs. A total of 1200 palm trees of the multi-parental population was planted in plots naturally infected by *Ganoderma*, and their health status was assessed biannually over 25 yr. The data were treated as survival data, and modeled using the Cox regression model, including a spatial effect to take the spatial component in the spread of *Ganoderma* into account. Based on the genotypes of 757 palm trees out of the 1200 planted, and on pedigree information, resistance loci were identified using a random effect with identity-by-descent kinship matrices as covariance matrices in the Cox model. Four *Ganoderma* resistance loci were identified, two controlling the occurrence of the first *Ganoderma* symptoms, and two the death of palm trees, while favorable haplotypes were identified among a major gene pool for ongoing breeding programs. This study implemented an efficient and flexible QTL mapping approach, and generated unique valuable information for the selection of oil palm varieties resistant to *Ganoderma* disease.

Plant diseases cause high yield losses for almost all crops worldwide, thus constituting a major food security constraint in a context of high population growth and shrinking cropland (Oerke and Dehne 2004). The genetics of plant resistance to pathogens is thus an active research area to support the selection of resistant crop varieties in the framework of integrated pest management programs geared toward limiting the use of chemical pesticides. R-genes controlling qualitative disease resistance have been identified in plants mainly by using biparental populations derived from crosses between resistant and susceptible parents (Gururani *et al.* 2012). Despite the efficiency of R-genes in the selection of resistant varieties, the sustainability of resistance could be improved by taking quantitative resistance loci into account (Poland *et al.* 2009), as shown empirically in studies on oilseed rape (Brun *et al.* 2010) and pepper (Quenouille *et al.* 2014). Broadening the genetic diversity screened using association panels is a way to identify such QTL (Wei *et al.* 2006; Adeyanju *et al.* 2015). Based on improved genetic and statistical properties (Verhoeven *et al.* 2005), multi-parental populations (MPPs) are increasingly being developed in many organisms (see the MPP collection http://www.genetics.org/content/multi-parental_populations), and their use for mapping quantitative resistance loci is promising (Kump *et al.* 2011; Benson *et al.* 2015; Cockram *et al.* 2015; Bajgain *et al.* 2016). Studies carried out in different contexts are needed to explore alternative experimental designs, appropriate statistical analyses, and to assess the potential of MPPs to gain insight into the genetic architecture of plant disease resistance.

Oil palm (*Elaeis guineensis* Jacq.) is a perennial allogamous species of African origin. It is currently the world’s leading fat resource, accounting for >30% of global vegetable oil production (USDA 2015), with a constantly increasing demand (FAOSTAT, http://www.fao.org/faostat/). Southeast Asia represents almost 90% of global production, but oil palm cultivation in this area is highly hampered by stem rot diseases, *i.e.*, basal (BSR) or upper (USR). BSR and USR are caused by *Ganoderma boninense*, a soil hemi-biotroph fungal pathogen that is also found in Africa and South America, where the disease is currently developing. *Ganoderma* disease is economically problematic when affecting ∼10–20% of palm trees, while 30–70% could be lost by the end of a typical 25-yr planting cycle, leading to substantial yield losses, premature replantation, and a waste of cropland (Durand-Gasselin *et al.* 2005; Cooper *et al.* 2011). The identification of genetic resistance in breeding material, based on field observations (de Franqueville *et al.* 2001) or screening tests with seedling inoculation (Idris *et al.* 2004; Breton *et al.* 2006), has led to the selection of partly resistant varieties. Information on the genetic architecture and molecular nature of *Ganoderma* resistance could help shorten the long oil palm breeding cycle (up to 20 yr), and enable its multi-criteria improvement using a marker-assisted selection strategy. All studies on *Ganoderma* disease to date have been based on seedling inoculation at the nursery stage: transcriptomic (Tee *et al.* 2013; Ho *et al.* 2016), proteomic (Al-Obaidi *et al.* 2014), or metabolomic (Nusaibah *et al.* 2016) approaches have identified genes, proteins, and pathways affected by *Ganoderma* infection, while an analysis based on 58 simple sequence repeat markers found alleles associated with *Ganoderma* symptoms in resistant *vs.* susceptible families (Hama-Ali *et al.* 2015). Field experiments are needed to assess broader genetic diversity in the agronomic context, but QTL analyses on oil palm crosses in the field are often not sufficiently effective because of population size and allelic segregation limitations (Jeennor and Volkaert 2014; Lee *et al.* 2014; Pootakham *et al.* 2015). Recent alternatives, based on data from breeding programs involving multiple connected families, have yielded promising results on agronomic traits (Billotte *et al.* 2010; Tisné *et al.* 2015).

In this study, an oil palm multi-parental population (*Eg*9PP) derived from nine founders of an ongoing breeding program was used to map *Ganoderma* disease resistance loci. Natural *Ganoderma* infection was monitored in the field, and the infection status of the 1200 *Eg*9PP individuals were recorded over 25 yr to generate a unique dataset. An innovative statistical modeling approach was implemented to map genetic loci affecting the survival of *Eg*9PP individuals, by combining a Cox regression model used for survival time QTL mapping in animal (Durrant *et al.* 2011; Gonen *et al.* 2015) or plant (Luo *et al.* 2013; Jiang *et al.* 2014) species, with an identity-by-descent (IBD)-based variance component approach (George *et al.* 2000) adapted to MPP design and with spatial effects modeling in order to control the confounding effects of *Ganoderma* epidemics. As *Eg*9PP parents are from genetic origins that are widely used in oil palm breeding programs, loci identified in this study provided highly relevant information to guide efficient marker-assisted selection for *Ganoderma* resistance.

## Materials and Methods

### Genetic material

**Figure 1:**
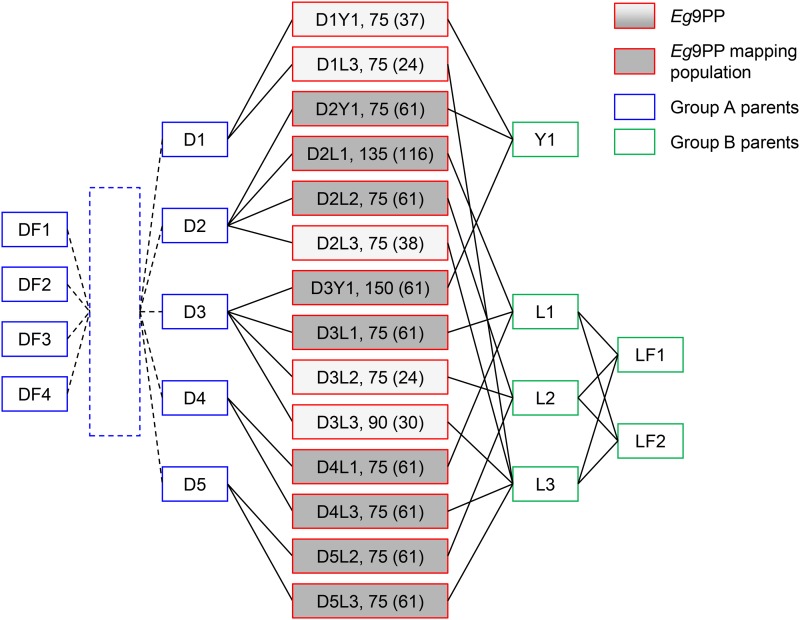
Pedigree of the *Eg*9PP oil palm multi-parental population. *Eg*9PP (red boxes, *n* = 1200) consisted of 14 full-sib families obtained by crossing parents from heterotic group A (blue boxes) and B (green boxes). The five parents from group A (D1–D5) derived from four founders (DF1–DF4) by partially known successive crosses and self-pollination (dotted lines). The number of individuals per family are indicated at the right side of the family name, with the number of sampled individuals in parenthesis (*n* = 757). Shaded boxes represent the fully genotyped families that constituted the mapping population (*n* = 604).

The oil palm (*Elaeis guineensis* Jacq.) multi-parental population (*Eg*9PP) was developed from 14 full-sib families generated by crossing five outbred parents of heterotic group A with four outbred parents of heterotic group B in an incomplete factorial design (Figure 1 and see Supplemental Material, Table S1). The group A parents belonged to the “Deli” population derived from four individuals planted in 1848 in Indonesia, and the group B parents belonged to “La Mé” (Cote d’Ivoire) or “Yangambi” (Democratic Republic of the Congo) populations (Corley and Tinker 2003). The *Eg*9PP parents are major founders of the PalmElit breeding program, a CIRAD subsidiary and leading oil palm breeding company (www.palmelit.com). This breeding program is shared and conducted with partners, *i.e.*, PT Socfin Indonesia (Indonesia) and INRAB (Benin). The 1200 *Eg*9PP individuals were planted in 1986 on the SOCFINDO estate (Medan, Indonesia), according to a randomized complete block design (RCBD) with at least five replications per family, each replication consisting of a plot with 15 full-sib individuals (see Figure S1). Despite the highly favorable agro-climatic conditions for oil palm growing, this location is hampered by the presence of *G. boninense*—the causal agent of basal stem rot disease that naturally infected *Eg*9PP.

Pedigree information was available for the La Mé parents, while the pedigree of the Deli parents was only known for a few (one or two) generations before the *Eg*9PP parents. The relatedness of the five Deli parents was estimated using pedigree reconstruction with MOLCOANC 3.0 software, as described in Cros *et al.* (2014), by using a larger Deli palm population (Tisné *et al.* 2015).

### Phenotype acquisition

The *G. boninense* infection status was recorded biannually on the 1200 *Eg*9PP individuals over 25 yr, from the first year after planting (June 1987) to the uprooting of the genetic trial (June 2012). Symptoms were scored blindly on the basis of a six level scale, set up in the 1980s, and used since that time in the SOCFINDO plantations, with zero being a healthy palm tree and six a dead and fallen palm tree with the presence of *Ganoderma* fruiting bodies on the palm trunk (see Figure S2 and Table S2). A seventh level represented palm trees absent at the visit time, or dead for reasons other than *Ganoderma* disease. Raw data were then curated, and the occurrence of two events was recorded with the time associated: the first *Ganoderma* symptom appearance (T1S, first observation of score 2–6), and the death of the palm tree due to *Ganoderma* disease (TD, first observation of score 6). Events of interest corresponded to T1S and TD, and the associated times were considered as survival times, *i.e.*, times from the start of the experiment to the time the event occurred (see Table S3 for phenotypic data). When the events were observed, the associated times were called exact or uncensored times, otherwise they were called censored observations.

### Molecular data and genetic map construction

A total of 757 individuals out of the 1200 from *Eg*9PP were sampled before uprooting the genetic trial because of premature death or illegitimacy (Figure 1). In a previous study, 116 individuals from the reference family D2L1 were genotyped with 390 single sequence repeat (SSR) markers, and a first reference high density linkage map grouping 255 SSRs and 688 amplified fragments length polymorphisms (AFLP) markers was published (Billotte *et al.* 2005). The SSR markers, plus 26 SSR additional markers previously mapped in a pedigree-based linkage analysis that was recently published (Cochard *et al.* 2015), were used to genotype 604 individuals from nine *Eg*9PP families, including D2L1, which constituted the mapping population (Figure 1). Based on genotyping of the *Eg*9PP parents, and the genetic position of SSR markers, different SSR marker subsets were used depending on the family, to optimize the genome coverage and marker information level (see Table S4 and Table S5 for individual genotypes). A QTL region identified in the linkage analysis of the mapping population (*n* = 604) was investigated in further detail by genotyping all of the 757 sampled individuals from the 14 *Eg*9PP families with all of the SSR markers mapped in the region plus two markers (see Table S4). DNA extraction, microsatellite amplification, and analyses were performed according to the protocol described in Cochard *et al.* (2015), and raw genotype data are available in Table S5.

The Eg9PP genetic map was constructed with CRI-MAP version 2.507 (Green *et al.* 1990). This program allows simultaneous analysis of multiple families and performs multipoint linkage analysis. The nine families of the *Eg*9PP mapping population were treated as unrelated families, and all individuals were considered as being of undetermined sex. Markers were grouped in separate input files based on the linkage group defined in the previously published genetic map (Billotte *et al.* 2005; Cochard *et al.* 2015). Markers were ordered in each linkage group using several runs of the BUILD command on the most informative markers, then the remaining markers were placed in the framework obtained using the ALL command. The FLIPS command was used to check alternative marker orders at the different linkage group construction stages, using different values up to four, depending on the number of markers and length of the linkage group analyzed. The FIXED option was finally used to calculate the Kosambi genetic distances between markers (see Figure S3 and Table S4). The genetic map obtained was drawn using MapChart software (Voorrips 2002).

We attempted to infer a physical position for each marker mapped using BLASTn (Zhang *et al.* 2000). We used forward and reverse primer sequences for each marker as query sequences against the oil palm genome sequence as the target database (Singh *et al.* 2013). We filtered the result hits of BLASTn with a minimum *e*-value threshold of 0.5. The marker position was assigned easily when forward and reverse primers were found in the same genome location. In case of multiple hits on different chromosomes for the same primer pair, the marker position was assigned based on the hit with the greatest *e*-value. Inferred physical positions of markers are available in Table S4.

Based on the marker physical positions, the physical positions of 210 predicted R-genes in the oil palm genome (Singh *et al.* 2013; Chan *et al.* 2017) were projected on the *Eg*9PP genetic map in order to identify colocalizations with *Ganoderma* resistance QTL (See Table S8).

### Linkage mapping

The linkage mapping of *Ganoderma* disease resistance was performed using a combination of survival data analyses and the two-step variance component approach described in George *et al.* (2000). The first step involved computation of IBD kinship matrices over a grid of genetic positions. The second step consisted of QTL presence tests over the grid of genetic positions by comparison of various models including random effect terms.

#### IBD matrix computation:

The variance component approach requires IBD kinship matrices as variance-covariance matrices for testing QTL effects at each genetic position. IBD analyses using SimWalk2 software (Sobel *et al.* 2001) were conducted to estimate the IBD matrices formed by empirical kinship coefficients for each pair of *Eg*9PP individuals and their parents. Simwalk2 implements a Bayesian framework using a Markov chain Monte Carlo (MCMC) algorithm to select the most likely genetic descent graph depicting inheritance patterns within pedigrees (Sobel and Lange 1996). Empirical kinship coefficients were first calculated between the 604 fully genotyped *Eg*9PP individuals and their parents at every marker position and over a 3 cM interval grid, leading to 1006 evaluation points for QTL presence testing (available at https://doi.org/10.6084/m9.figshare.4780489.v1).

#### Statistical modeling:

The Cox regression model (Cox 1972) is a popular choice for analyzing the relationship between the survival outcome and explanatory covariates X. This semiparametric model has the following hazard function:

$$\lambda \left(t,\;X\right)=\;\lambda_{0}\left(t\right)e^{X\;\beta },\;$$ (1)

where $\beta^{\prime }=\left(\beta_{1},\ldots ,\beta_{m}\right)$ is a m×1 unknown vector of parameters reflecting the effects of covariates $X^{\prime }=\left(X^{1},\ldots ,X^{m}\right)$ on survival, t is the time to the event or censoring, and $\lambda_{0}$ denotes the baseline hazard function. Note that no particular shape was assumed for the baseline function.

In this study, the analysis of data based on natural field infection required taking into account the spatial component of the propagation of *Ganoderma* disease. The spatial (plot) information was included to model (1), as well as genetic (family and individual) information to avoid the genetic variance being absorbed by spatial variance due to the experimental design. One way of considering this information is to extend the model (1) to a mixed effect Cox model by adding one random effect with a covariance matrix based on pedigree, and one with a spatially structured covariance matrix based on the geographic coordinates of the plot. Vaupel *et al.* (1979) were the first to introduce random effects (called frailties) in the survival model in order to group time-to-event data within *clusters*.

Model (2) is then defined as follows:

$$\lambda \left(t,\;X\right)=\lambda_{0}\left(t\right)e^{X\beta +Z_{1}u+Z_{2}g},$$ (2)

with n corresponding to the number of individuals, X∈n×P is the design matrix relating the observations to the P×1 vector of family fixed effects, $Z_{1}\in n\times S$ the design matrix relating the survival outcome to the S×1 vector of spatial random effects $u={\left(u_{1},\ldots ,u_{S}\right)}^{\prime }$ at the plot level, and $Z_{2}\in n\times \left(n+m\right)$ is the design matrix relating the survival outcome to the additive genetic random effects $g={\left(g_{1},\ldots ,g_{n+m}\right)}^{\prime }$ with m the number of parents.

Concerning the spatial random effects, a conditionally auto-regressive (CAR) structure (Cressie 1993) was used in order to model dependence among observations in neighboring plots (Banerjee *et al.* 2003). Where N(s) denotes the neighborhood of plot s including the plots which share a common boundary, the distribution of u is a multivariate Normal distribution defined by:

$$u∼\mathrm{MVNormal}\left(0,\sigma_{u}^{2}{\left(D-\alpha W\right)}^{-1}\right),$$

where α is the spatial autocorrelation parameter, W the adjacency matrix (*i.e.*, $W_{ss}=0,$ $W_{ss^{\prime }}=1$if *s* and *s*′ are neighbors, and $W_{ss^{\prime }}=0$ otherwise), $\sigma_{u}^{2}$ is the spatial dispersion parameter, and *D* is a diagonal matrix with diagonal elements equal to $N_{s},$ *i.e.*, the number of neighbors for the plot *s*. The additive genetic random effects also follow a multivariate Normal distribution:

$$g∼\mathrm{MVNormal}\left(0,\;\;\sigma_{a}^{2}A\right),$$

with A∈(n+m)×(n+m)being the kinship matrix based on pedigree information, and $\sigma_{a}^{2}$ the additive genetic variance.

To save computation time in the QTL mapping step, the model (2) was used for predicting spatial random effects associated with each plot, and the spatial effects predictions $u={\left(u_{1},\ldots ,u_{S}\right)}^{\prime }$ were included as fixed effects in the following QTL models (see Table S7 for model (2) results). The model considered as null model for QTL mapping was then defined by:

$$\lambda \left(t,\;X\right)=\lambda_{0}\left(t\right)e^{X\beta }$$ (3)

where X is the $n_{g}\times \left(P+1\right)$ design matrix relating the survival outcome for genotyped individuals to fixed effects (family and spatial effects). The null model defined in (3) was compared to the model (4) such that:

$$\lambda \left(t,\;X\right)=\lambda_{0}\left(t\right)e^{X\beta +Z_{q}q^{p}}$$ (4)

with $Z_{q}\in n_{g}\times \left(n_{g}+m\right)$ being the design matrix relating the survival outcome for genotyped individuals to the QTL random effect $q^{p}={\left(q_{1}^{p},\ldots ,q_{n_{g}+m}^{p}\right)}^{\prime }$ at the *p*th position on the genome. The QTL random effect at the *p*th position $q^{p}$ followed a multivariate normal distribution, with a variance-covariance matrix equal to the IBD kinship matrix $M^{p}$ at the *p*th position then being $q∼\mathrm{MVNormal}\left(0,\sigma_{q^{p}}^{2}M^{p}\right).$

A genome scan was performed to test the QTL presence by computing the log-likelihood ratio to compare models (3) and (4) at each p position. Best linear unbiased predictions (BLUPs) of QTL effect were computed for each *Eg*9PP individual using model (4) at the retained genetic positions (see next paragraph).

#### Log-likelihood ratio test:

The log-likelihood ratio test (LRT) was used to test whether frailty (QTL) variance was null at each p position. With a non-negative restriction on the variance component, the LRT is one-sided and has null value on the boundary of parameter space, so the classical likelihood ratio asymptotic chi-squared distribution theory is no longer valid. The distribution of LRT is then unknown under the null hypothesis H_0_, so an empirical approach by permutations (Churchill and Doerge 1994) and a theoretical approach were used to estimate significance levels. In the permutation approach, different LRT significance levels were calculated for each putative QTL based on 500 chromosome-wide permutations obtained by shuffling the identity of individuals in the data to break genotype-phenotype associations. For each permutation, the maximum LRT value obtained was recorded to construct the distribution of the maximum LRT value under the null hypothesis (see Figure S4A). The empirical type I error rates associated with each LRT distribution were calculated so as to estimate the significance level of each LRT peak detected (see Figure S4B). The theoretical approach was based on different works in shared frailty model framework (Duchateau *et al.* 2002; Vu and Knuiman 2002; Claeskens *et al.* 2008) that assessed the limiting distribution of LRT. The resulting distribution is the asymptotic 50:50 mixture of the $\chi_{0}^{2}$ and the $\chi_{1}^{2}$ chi-squared distributions of 0 and 1 degree of freedom, respectively, that was used to calculate *p*-values associated to each QTL detected.

#### Haplotype analysis:

QTL mapping identified a strong QTL in the top of linkage group 1, and this region was analyzed in further detail. Based on the genotyping of all the 757 sampled individuals from the 14 *Eg*9PP families with seven SSR markers covering 20 Mb (see Table S4), the kinship coefficients were calculated between the 757 *Eg*9PP individuals and used to perform QTL mapping with this extended dataset in order to determine precise QTL location in the candidate region. Then, haplotype analysis was performed in the candidate region using SimWalk2 to identify the funder allele segregation in the Eg9PP individuals. Funder allele effects were then estimated including two fixed effects in model (3)—one for each heterotic group—in order to identify favorable alleles among the *Eg*9PP parents at the QTL peak closest marker.

All analyses were performed with R software version 3.2.3 (R Development Core Team 2012) and the coxme package (Therneau and Therneau 2015), and R scripts, with example data files and documentation, are available at the github repository https://github.com/DenisMarie/Eg9PP_Ganoderma. The genome scan results are available in Table S6.

### Data availability

*Eg*9PP was uprooted in 2012, but *Eg*9PP families can be reproduced by crossing individuals derived from the self-fertilized progenies of *Eg*9PP parents. Requests for such genetic material should be addressed to PalmElit (www.palmelit.com). Raw genotype data are available in Table S5 and IBD kinship matrices at https://doi.org/10.6084/m9.figshare.4780489.v1. Phenotyping of disease events are available in Table S3. All R scripts, data file and documentation are available at https://github.com/DenisMarie/Eg9PP_Ganoderma.

## Results

### Variations in Ganoderma resistance in Eg9PP

**Figure 2:**
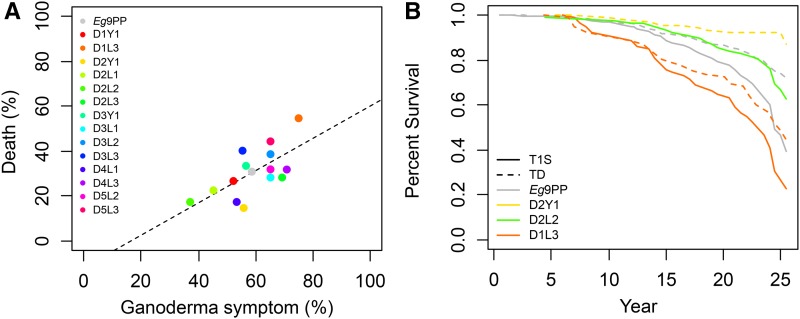
*Ganoderma* disease epidemics in the *Eg*9PP oil palm multi-parental population. (A) Relationship between the percentages of affected and dead individuals per family at the trial uprooting. Colors corresponding to families are indicated in the legend at the top left, with gray circles indicating the *Eg*9PP value (*n* = 1200). Dotted lines represent the linear regression between the percentages of affected and dead individuals (slope = 0.72, Pearson correlation coefficient *r* = 0.66, *p*-value = 0.009). (B) Survival curves for *Eg*9PP and *Eg*9PP families exhibiting maximum (D1L3) and minimum (D2Y1, D2L2) percentages of affected and dead individuals at the trial uprooting (color legend at bottom left). Survival estimates are plotted for two events, the time of the first *Ganoderma* symptom observation (T1S, solid lines), and death due to *Ganoderma* infection (TD, dotted lines).

*Ganoderma* disease resistance was assessed in 1200 *Eg*9PP individuals by biannually recording their infection status over 25 yr. At the end of the experiment, 58.5% of the individuals exhibited *Ganoderma* symptoms over this period, and 30.5% died due to *Ganoderma* disease (Figure 2A and see Table S7). These percentages ranged from 74.7 and 54.7% for infected and dead individuals, respectively, for the worst family D1L3, to 37.3 and 14.7% for the best families D2L2 and D2Y1 (Figure 2A and see Table S7). The percentages of individuals per family infected and dead at the uprooting of the trial were significantly correlated (*r* = 0.66, Figure 1A), but families with a similar percentage of infected individuals exhibited a substantially different percentage of death (*e.g.*, D3L3 and D2Y1, Figure 2A). The times of the first *Ganoderma* symptom appearance (T1S) and death due to *Ganoderma* (TD) were modeled using Cox regression including spatial and family effects, and both effects were found to be highly significant (*P* < 0.001). Risks per family ranged from 0.32 to 0.98 for T1S, corresponding to 3.1-fold lower risk for the best family (D2L2) compared to the worst family (D1L3) (Figure 1B and see Table S7). For TD, risks per family ranged from 0.2 to 0.96, corresponding to 4.9-fold lower risk for the best family (D2Y1) compared to the worst family (D1L3) (Figure 1B and see Table S7). In addition to this interfamily variance, large confidence intervals for risk per family indicated other sources of variance that could be captured by QTL effects in families (see Table S7). Kaplan-Meier estimates of survival showed that the economically significant threshold of 20% affected palm trees was reached 15 yr after planting for the worst *Eg*9PP family as compared to 24 yr for the best (Figure 1B).

### Linkage mapping of Ganoderma resistance loci

**Figure 3:**
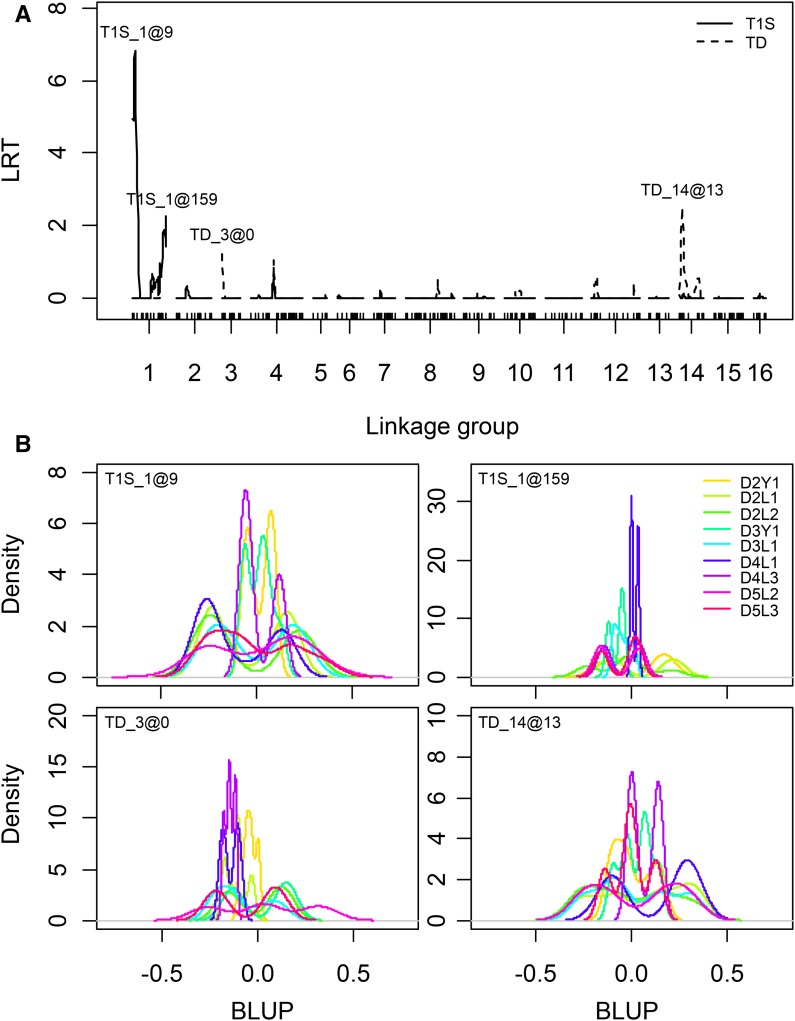
Genetic determinism of *Ganoderma* resistance in the *Eg*9PP oil palm multi-parental population. (A) LRT testing the presence of a QTL for the time of the first *Ganoderma* symptom observation (T1S, solid lines) and death due to *Ganoderma* infection (TD, dotted lines) in the *Eg*9PP mapping population (*n* = 604). Dashes on the *x*-axis represent markers on the genetic map. Labels above LRT peaks (Trait_Linkage group@position in cM) indicate QTL with an LRT below a type I error rate of 0.5. (B) BLUP distributions for random QTL effects (QTL names at top left of each panel) for the *Eg*9PP families of the mapping population (*n* = 604, color legend at top right).

QTL analysis in the *Eg*9PP mapping population (*n* = 604) identified two regions associated with the time of the first symptom observation (T1S), and two with the time of death due to *Ganoderma* infection (TD), with no overlap between them (Figure 3A). The inclusion of spatial effects in the base model did not change the QTL results for T1S despite the reduced overall LRT level (see Figure S5). On the contrary, three LRT peaks for TD were lost by adding the spatial effect (middle of LG1 and LG4, bottom of LG14), but a peak at the top of LG3 was increased (see Figure S5). The strongest QTL was found for T1S at the top of LG1, with an LRT of 6.83 at a significance level (*α*) of 0.01 (T1S_1@9, Table 1). The variance associated with T1S_1@9 was 0.16, corresponding to a risk $\exp\left(\sqrt{0.16}\right)$ = 1.49-fold above the norm for ∼15% of the individuals, with a similar fraction of individuals having lower risk. The distribution of individual BLUP indicated the greatest risk segregation in families from La Mé parents L1, L2, and L3 (Figure 3B). The second QTL for T1S (T1S_1@159) was detected at the bottom on LG1, with a risk of 1.30 (*α* = 0.29), with risk segregation found mainly in families from parent D2 (Figure 3B and Table 1). Both QTL detected for TD, TD_3@0, and TD_14@13 at the top of LG3 and LG14, respectively, had high risks (1.45) but low significance levels (*α* = 0.38 and 0.19, respectively), with a risk BLUP distribution indicating the greatest QTL segregation in families from parents D3 and D5 for TD_3@0, and L1 and L2 for TD_14@13 (Figure 3B and Table 1).

**Table 1:** QTL associated with *Ganoderma* resistance in the *Eg*9PP oil palm multi-parental population

Traits	QTL*^a^*	LRT*^b^*	α*^c^*	*p*-value*^d^*	Variance	Closest Marker	Chromosome*^e^*	Physical Position*^f^*
T1S								
	T1S_1@9	6.83	0.01	0.004	0.16	mEgCIR3803	3	58.17
	T1S_1@159	2.24	0.29	0.067	0.07	mEgCIR0332	3	—
TD								
	TD_3@0	1.23	0.38	0.134	0.14	mEgCIR3698	8	30.15
	TD_14@13	2.50	0.19	0.057	0.14	mEgCIR2427	11	23.86

a QTL name indicates the trait, the number of linkage group and the peak position on the genetic map in cM.

b LRT Log-likelihood ratio test.

c Empirical type I error rate determined by permutation tests.

d *p*-values computed from a 50:50 mixture of the $\chi_{0}^{2}$ and the $\chi_{1}^{2}.$

e Chromosome number according to Singh *et al.* (2013).

f Physical position of the closest marker in Mb.

### Haplotype analysis of the major Ganoderma resistance QTL

**Figure 4:**
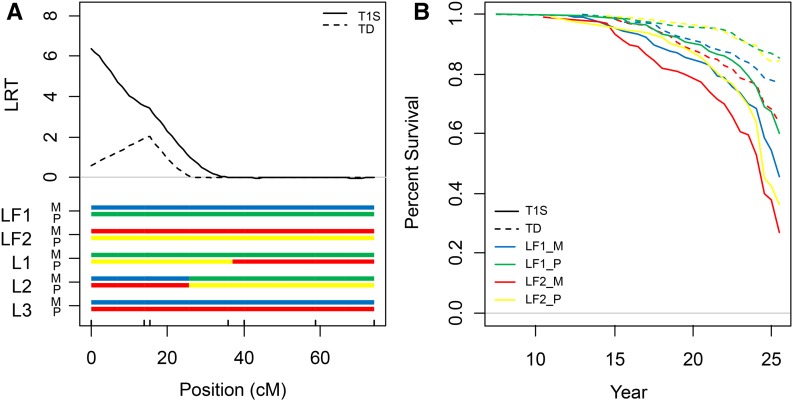
Haplotype analysis of *Ganoderma* resistance QTL T1S_1@9. (A) LRT testing the presence of a QTL at the top of linkage group 1 for the time of the first *Ganoderma* symptom observation (T1S, solid lines) and death due to *Ganoderma* infection (TD, dotted lines) in the *Eg*9PP sampled individuals (*n* = 757). Dashes on the *x*-axis represent markers on the genetic map. La Mé founder (LF1, LF2), and parent (L1 to L3) maternal (M) and paternal (P) IBD alleles are represented by different colors below the LRT lines. (B) Survival curves for the *Eg*9PP sampled individuals (*n* = 757) according to the IBD allele they inherited from their La Mé parent. Survival estimates are plotted for two events, *i.e.*, the time of the first *Ganoderma* symptom observation (T1S, solid lines), and death due to *Ganoderma* infection (TD, dotted lines).

T1S_1@9, the major QTL for *Ganoderma* resistance, was investigated in further detail by genotyping all sampled *Eg*9PP individuals (*n* = 757) with the five SSR markers in the region (LG1, 0–44.5 cM), by adding two additional SSR markers and performing IBD and haplotype analyses. IBD-QTL mapping on the augmented dataset confirmed the presence of a QTL for T1S on LG1, but the LRT peak position shifted to 0 cM on the mEgCIR1713 marker (Figure 4A). While no QTL were found for TD in this region, a LRT peak was detected with the augmented dataset close to the mEgCIR3803 marker (Figure 4A). Cox regression models including two fixed effects for haplotypes at the closest markers in each heterotic group indicated significant effects in group A (*p*-value < 0.01) and group B (*p*-value < 0.001) for T1S as well as for TD (*p*-values < 0.01 and < 0.001 for groups A and B, respectively). The four La Mé founder IBD alleles were present in this region in the three La Mé *Eg*9PP parents, with two recombination events in the T1S_1@9 region in founder meiosis (Figure 4A). Marked differences in estimated risk were observed for individuals carrying alternative La Mé founder haplotypes: individual with an LF1 paternal allele (LF1_P, Figure 4B) had a 2.1-fold lower risk of being infected by *Ganoderma* and 2.07-fold lower risk of dying due to *Ganoderma* compared to the worst allele (LF2_M). The survival estimates showed that the 20% affected palm tree threshold was reached 19.5 yr after planting for individuals carrying the LF2_M allele, as compared to >23.5 yr for those carrying the LF1_P allele (Figure 4B). Moreover, this haplotype analysis allowed us to identify favorable alleles within the Yangambi and Deli genetic backgrounds associated with contrasted risks of *Ganoderma* infection and death (see Figure S6).

## Discussion

This study was based on data from monitoring natural field infection of an oil palm multi-parental population over 25 yr. This unique dataset provided an opportunity to map *Ganoderma* resistance loci in natural conditions according to an economic and efficient *in silico* approach, *i.e.*, based on data from breeding programs (Parisseaux and Bernardo 2004). Four *Ganoderma* resistance loci were identified by combining different statistical methods, including survival analysis, infection spatial modeling, and a variance component approach for QTL mapping.

### Disease resistance loci mapping using the Cox regression model

The QTL mapping approach was developed in the framework of normally distributed quantitative traits. In this study, *Ganoderma* infection data were treated as survival traits, with censoring because of unobserved events. Such data are commonly found in human or animal studies in which most of the statistical methods were developed. Zhao (2005) proposed a statistical approach using a mixed-effects Cox model to study familial effects in alcohol dependence. In salmon, disease resistance loci were mapped in complex populations using a variance component approach on binary survival data (Gonen *et al.* 2015). In plants, especially annual ones, survival analyzes are seldom performed because of the experimental potential, but methods have been developed to map QTL associated with flowering time in biparental rice (Luo *et al.* 2013) and barley (Jiang *et al.* 2014) populations. As suggested in Zhao (2005), QTL mapping was performed in this study using IBD kinship matrices, calculated with Simwalk2 (Sobel *et al.* 2001), as covariance matrices of the QTL random effect. This approach is flexible in terms of population design, and was found to be efficient in a former study on agronomic quantitative traits in a complex oil palm pedigree using a linear mixed effect model (Tisné *et al.* 2015). Modeling of a spatial effect to account for the variance associated with the natural inoculation of the palm trees was found to be highly significant, and changed the time of death mapping results. However, it is unclear whether it improved the mapping results without additional information, *e.g.*, from simulation studies. Further developments could improve the mapping method. Modeling QTL effects including two variance components, one for each heterotic group, would enable independent testing of the QTL presence in each genetic background and assessing the importance of nonadditive effects, *i.e.*, dominance or epistasis. A multi-QTL approach would also be worthwhile as the multiple QTL methods proved their superiority in estimating QTL effects and position (Jansen 1993). This could be achieved using a variable selection approach in the Bayesian framework, as developed for quantitative traits (Bink *et al.* 2008).

The approach described in this study—flexible, efficient, with further possible extensions—could be interesting for studying disease resistance in plants, especially for perennials, which have limited experimental potential.

### Genetic architecture of Ganoderma disease resistance

Plant disease resistance is commonly partitioned between R-gene mediated qualitative or complete resistance, and quantitative or incomplete resistance controlled by many quantitative resistance loci (Poland *et al.* 2009). In the present study, no QTL were found that completely prevented *Ganoderma* infection or death, which is consistent with field observations that could not highlight such complete resistance (Durand-Gasselin *et al.* 2005). Colocalizations of *Ganoderma* resistance QTL with oil palm predicted R-genes were observed (see Table S8), but further work would be needed to validate these genes as candidate. Moreover, R-gene mediated complete resistance was observed almost exclusively with biotrophic pathogens, so it is unlikely that such resistance occurs in oil palm, with *Ganoderma* being an hemi-biotrophic pathogen (Ho and Tan 2015). However, marked delayed effects on the time of the first symptom observation (T1S) and the time of death (TD) were observed among families (threefold to fivefold, respectively), with high variance associated with QTL noted for both T1S and TD. The different QTL patterns observed for T1S and TD, without any colocalizations noted, except at the top of LG1, was a striking result. This is consistent with the correlation at the family level between T1S and TD being looser than expected, indicating variation in the survival time when infected by *Ganoderma*. This could still be linked to the two stages of infection reported for *Ganoderma*, *i.e.*, biotrophic then necrotrophic, as it is known that both induce different immune responses that could lead to different genetic architectures for T1S and TD. Poland *et al.* (2009) put forward six hypotheses on the mechanisms underlying quantitative resistance loci, with the first being the effect of pleiotropic genes regulating morphological and developmental phenotypes. Mapping of yield and morphology traits in *Eg*9PP (unpublished results) revealed colocalization of TD QTL with fruit weight and composition (TD_3@0) and stem height (TD_14@13), indicating possible pleiotropic effects as a basis of quantitative resistance to *Ganoderma*.

This is the first study that identified the genetic determinism of *Ganoderma* resistance in the field, so the detected QTL need further validation in independent experiments, accompanied by a fine mapping approach to identify the underlying genes. Despite a marker density suitable for QTL mapping (one marker every 8 cM on average), the analysis of *Eg*9PP would benefit from denser marker sets, *e.g.*, SNP obtained by genotyping-by-sequencing (Pootakham *et al.* 2015) or high density genotyping array (Kwong *et al.* 2016) that were recently developed in oil palm. Haplotype analysis of the T1S_1@9 region exhibited many recombination events (105 in 1.9 Mb), indicating that densifying *Eg*9PP genotyping in the candidate regions could lead to candidate gene identification. *Eg*9PP individuals were uprooted but leaf samples are still available, and acquisition of a denser genotyping is in progress. Although field experiments are certainly more relevant, nursery tests with inoculation are interesting to accumulate genetic and omic knowledge on *Ganoderma* resistance. Unfortunately, the only nursery-based genetic study to date (Hama-Ali *et al.* 2015) showed no overlap between field resistant QTL and SSR markers associated with *Ganoderma* disease incidence in the nursery despite some common markers and genetic backgrounds (Deli × Yangambi). The scope of the study, *i.e.*, involving 79 individuals from one tolerant and two susceptible families, genotyped with 58 SSRs, made it difficult to conclude on the transferability of the QTL results from the nursery to the field, and further research is needed to map resistance QTL based on nursery inoculated populations.

### Contribution to Ganoderma resistance loci in oil palm breeding programs

The multi-parental population design allowed us to identify quantitative resistant loci among an extended genetic diversity, and to test their effects in various genetic backgrounds, which should enhance the transferability of results and the sustainability of the selected resistances. The most widely used oil palm breeding scheme was developed in the 1950s from a reciprocal recurrent selection (RRS) scheme to take advantage of heterotic effects on bunch production observed between group A and B genetic backgrounds, represented by Deli and La Mé/Yangambi parents, respectively, in the present study (Gascon and de Berchoux 1964). Despite substantial differences in *Ganoderma* resistance levels among the three genetic origins assessed, with Deli being highly susceptible in comparison to Yangambi and La Mé material (Durand-Gasselin *et al.* 2005), allelic segregation was found in the Deli background for the major QTL T1S_1@9 (D1 and D5 parents), QTL T1S_1@159 (D2 parent), and QTL TD_3@0 (D3 and D5 parents). Hence, RRS would be efficient for both heterotic groups by selecting quantitative resistant alleles at different loci within genetic origins and combining them in A × B producer hybrids.

Localization of *Ganoderma* resistance loci will facilitate multi-criteria improvement of oil palm, with the possibility of combining known favorable genomic regions, *e.g.*, for disease and yield related traits, while avoiding unintended unfavorable selection on the others. This is currently feasible since an increasing number of studies have reported QTL for agronomic traits such as bunch production and sex ratio (Ukoskit *et al.* 2014; Tisné *et al.* 2015), mesocarp oil content (Teh *et al.* 2016), fatty acid composition (Montoya *et al.* 2014), stem height (Lee *et al.* 2014), and lipase activity (Morcillo *et al.* 2013). The selection of varieties resistant to a combination of diseases will be essential for this multi-criteria improvement, as *Ganoderma* is found throughout the oil palm cultivation area, combined with other devastating diseases like the *Fusarium* wilt in Africa, and bud rot in South America. Hence, the global genetic architecture of disease resistance in oil palm needs to be investigated, especially potential trade-off effects related to different pathogens, as shown for other species (Poland *et al.* 2009). The multi-parental population presented in this study, and the implemented QTL mapping approach, provide powerful tools for investigating such global genetic architecture of disease resistance in oil palm.

## Supplementary Material

Supplemental material is available online at http://www.g3journal.org/lookup/suppl/doi:10.1534/g3.117.041764/-/DC1.
