# Supplementary material for: Identification of Ganoderma Disease Resistance Loci Using Natural Field Infection of an Oil Palm Multiparental Population
Source: G3 (Bethesda). 2017 Jun 5;7(6):1683–92. doi: 10.1534/g3.117.041764 (PMC5473749; doi:10.1534/g3.117.041764)
Supplement: Supplementary file 3 [file 1683File003.pptx]

## Slide 1
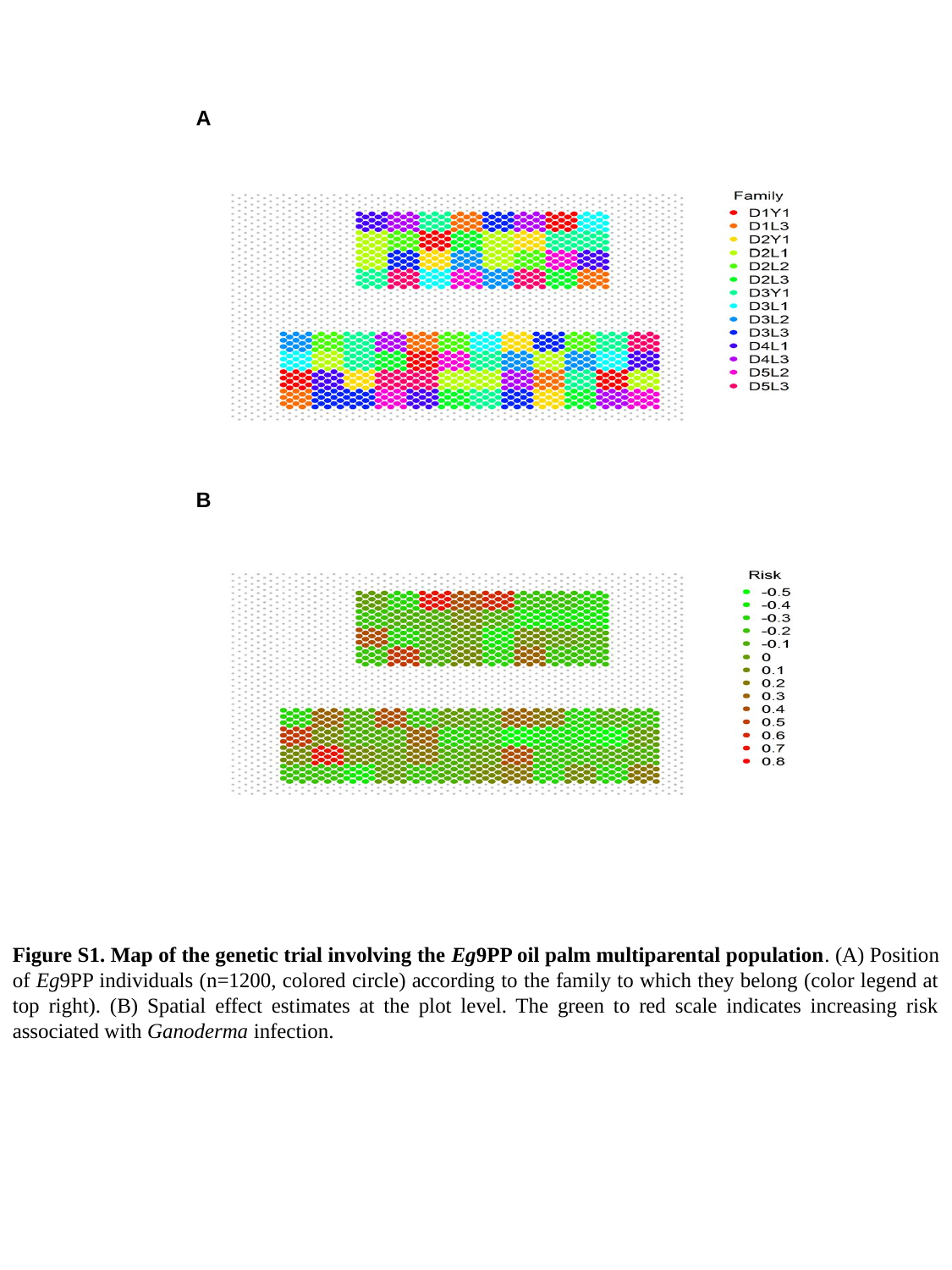

A
B
Figure S1. Map of the genetic trial involving the Eg9PP oil palm multiparental population. (A) Position of Eg9PP individuals (n=1200, colored circle) according to the family to which they belong (color legend at top right). (B) Spatial effect estimates at the plot level. The green to red scale indicates increasing risk associated with Ganoderma infection.

## Slide 2
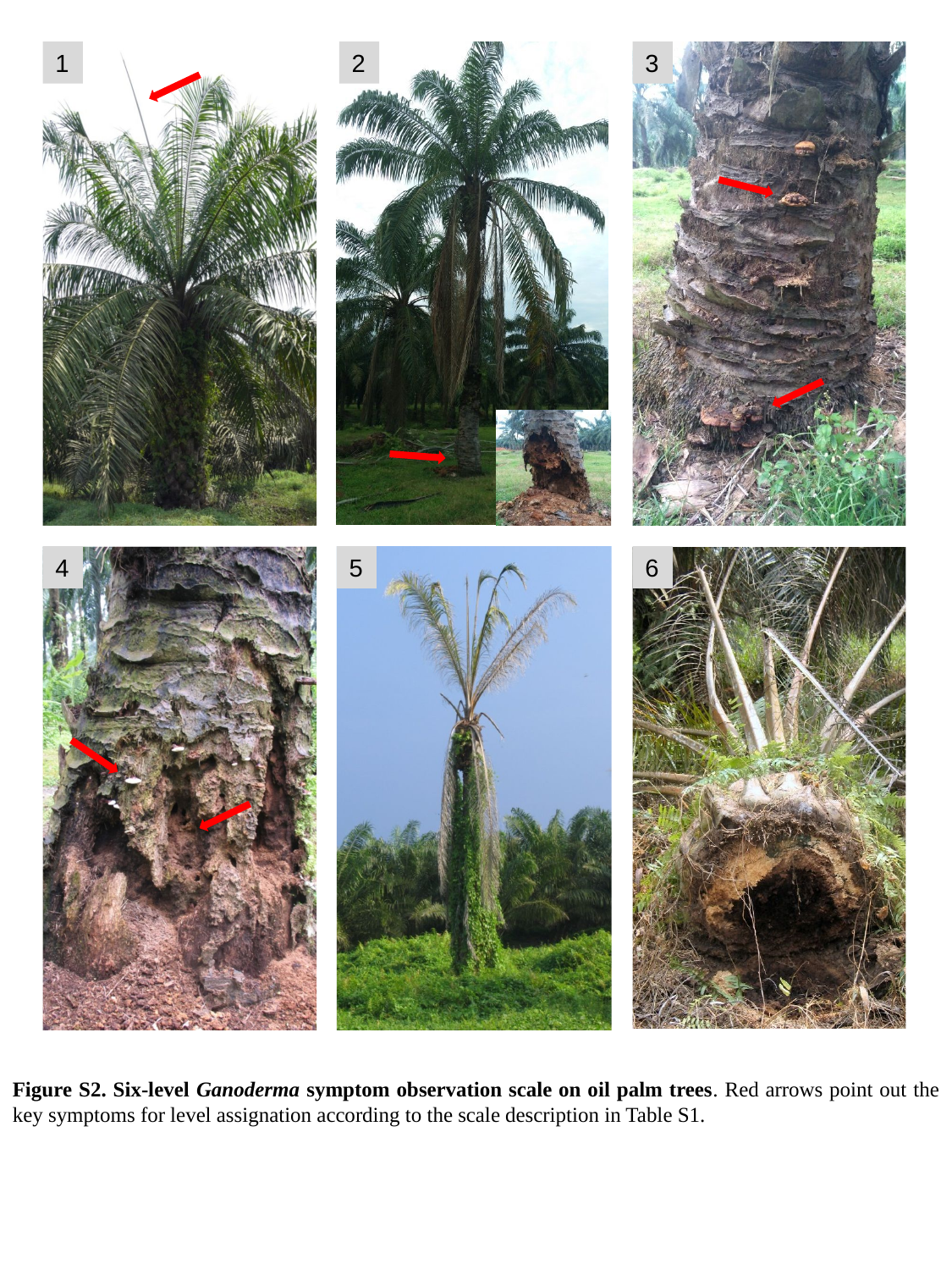

1
2
3
4
5
6
Figure S2. Six-level Ganoderma symptom observation scale on oil palm trees. Red arrows point out the key symptoms for level assignation according to the scale description in Table S1.

## Slide 3
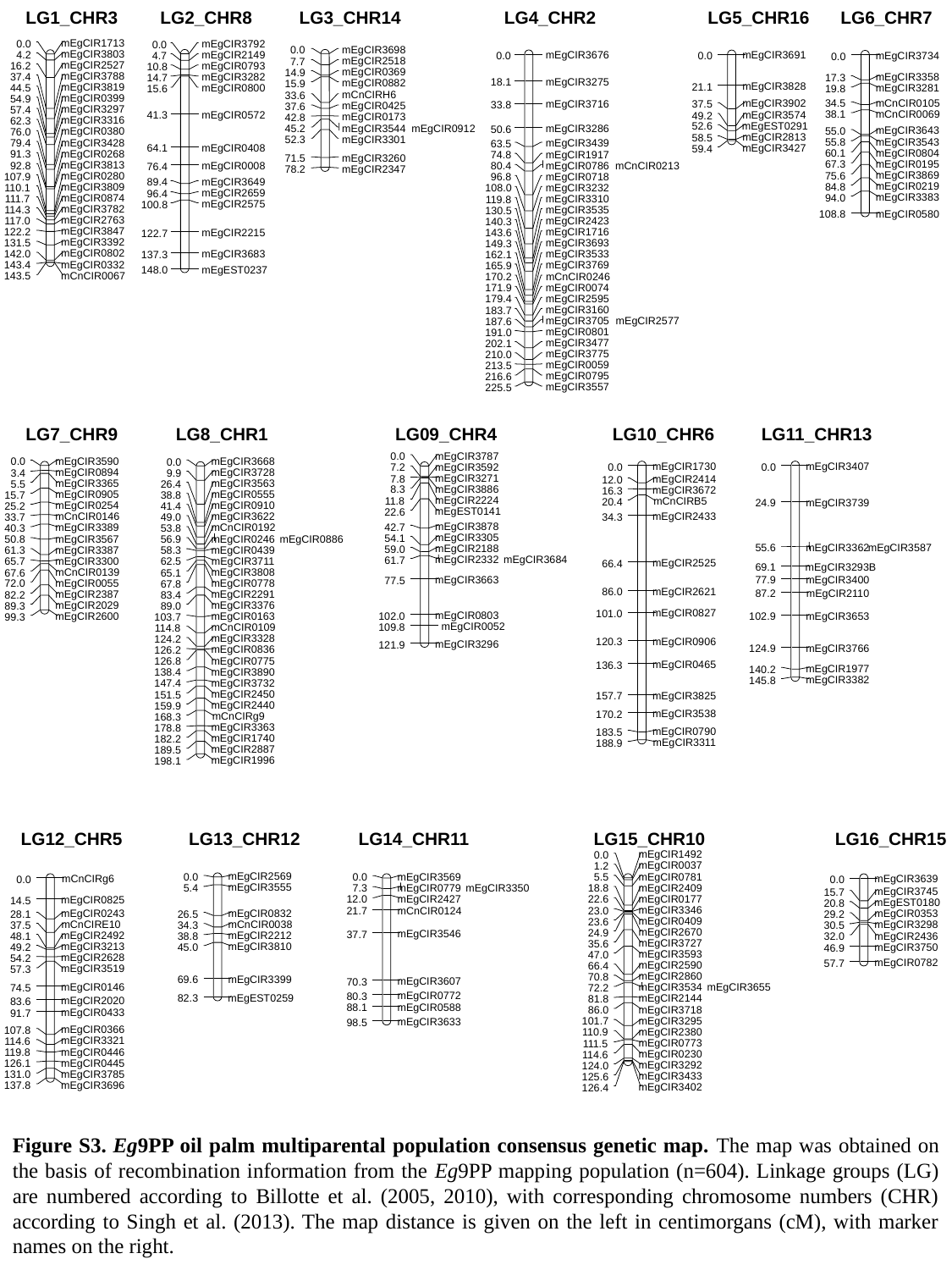

LG1_CHR3
LG2_CHR8
LG3_CHR14
LG4_CHR2
LG5_CHR16
LG6_CHR7
mEgCIR1713
0.0
mEgCIR3803
4.2
mEgCIR2527
16.2
mEgCIR3788
37.4
mEgCIR3819
44.5
mEgCIR0399
54.9
mEgCIR3297
57.4
mEgCIR3316
62.3
mEgCIR0380
76.0
mEgCIR3428
79.4
mEgCIR0268
91.3
mEgCIR3813
92.8
mEgCIR0280
107.9
mEgCIR3809
110.1
mEgCIR0874
111.7
mEgCIR3782
114.3
mEgCIR2763
117.0
mEgCIR3847
122.2
mEgCIR3392
131.5
mEgCIR0802
142.0
mEgCIR0332
143.4
mCnCIR0067
143.5
mEgCIR3792
0.0
mEgCIR2149
4.7
mEgCIR0793
10.8
mEgCIR3282
14.7
mEgCIR0800
15.6
mEgCIR0572
41.3
mEgCIR0408
64.1
mEgCIR0008
76.4
mEgCIR3649
89.4
mEgCIR2659
96.4
mEgCIR2575
100.8
mEgCIR2215
122.7
mEgCIR3683
137.3
mEgEST0237
148.0
mEgCIR3698
0.0
mEgCIR2518
7.7
mEgCIR0369
14.9
mEgCIR0882
15.9
mCnCIRH6
33.6
mEgCIR0425
37.6
mEgCIR0173
42.8
mEgCIR3544
mEgCIR0912
45.2
mEgCIR3301
52.3
mEgCIR3260
71.5
mEgCIR2347
78.2
mEgCIR3676
0.0
mEgCIR3275
18.1
mEgCIR3716
33.8
mEgCIR3286
50.6
mEgCIR3439
63.5
mEgCIR1917
74.8
mEgCIR0786
mCnCIR0213
80.4
mEgCIR0718
96.8
mEgCIR3232
108.0
mEgCIR3310
119.8
mEgCIR3535
130.5
mEgCIR2423
140.3
mEgCIR1716
143.6
mEgCIR3693
149.3
mEgCIR3533
162.1
mEgCIR3769
165.9
mCnCIR0246
170.2
mEgCIR0074
171.9
mEgCIR2595
179.4
mEgCIR3160
183.7
mEgCIR3705
mEgCIR2577
187.6
mEgCIR0801
191.0
mEgCIR3477
202.1
mEgCIR3775
210.0
mEgCIR0059
213.5
mEgCIR0795
216.6
mEgCIR3557
225.5
mEgCIR3691
0.0
mEgCIR3828
21.1
mEgCIR3902
37.5
mEgCIR3574
49.2
mEgEST0291
52.6
mEgCIR2813
58.5
mEgCIR3427
59.4
mEgCIR3734
0.0
mEgCIR3358
17.3
mEgCIR3281
19.8
mCnCIR0105
34.5
mCnCIR0069
38.1
mEgCIR3643
55.0
mEgCIR3543
55.8
mEgCIR0804
60.1
mEgCIR0195
67.3
mEgCIR3869
75.6
mEgCIR0219
84.8
mEgCIR3383
94.0
mEgCIR0580
108.8
LG7_CHR9
LG8_CHR1
LG09_CHR4
LG10_CHR6
LG11_CHR13
mEgCIR3787
0.0
mEgCIR3592
7.2
mEgCIR3271
7.8
mEgCIR3886
8.3
mEgCIR2224
11.8
mEgEST0141
22.6
mEgCIR3878
42.7
mEgCIR3305
54.1
mEgCIR2188
59.0
mEgCIR2332
mEgCIR3684
61.7
mEgCIR3663
77.5
mEgCIR0803
102.0
mEgCIR0052
109.8
mEgCIR3296
121.9
mEgCIR3590
0.0
mEgCIR0894
3.4
mEgCIR3365
5.5
mEgCIR0905
15.7
mEgCIR0254
25.2
mCnCIR0146
33.7
mEgCIR3389
40.3
mEgCIR3567
50.8
mEgCIR3387
61.3
mEgCIR3300
65.7
mCnCIR0139
67.6
mEgCIR0055
72.0
mEgCIR2387
82.2
mEgCIR2029
89.3
mEgCIR2600
99.3
mEgCIR3668
0.0
mEgCIR3728
9.9
mEgCIR3563
26.4
mEgCIR0555
38.8
mEgCIR0910
41.4
mEgCIR3622
49.0
mCnCIR0192
53.8
mEgCIR0246
mEgCIR0886
56.9
mEgCIR0439
58.3
mEgCIR3711
62.5
mEgCIR3808
65.1
mEgCIR0778
67.8
mEgCIR2291
83.4
mEgCIR3376
89.0
mEgCIR0163
103.7
mCnCIR0109
114.8
mEgCIR3328
124.2
mEgCIR0836
126.2
mEgCIR0775
126.8
mEgCIR3890
138.4
mEgCIR3732
147.4
mEgCIR2450
151.5
mEgCIR2440
159.9
mCnCIRg9
168.3
mEgCIR3363
178.8
mEgCIR1740
182.2
mEgCIR2887
189.5
mEgCIR1996
198.1
mEgCIR1730
0.0
mEgCIR2414
12.0
mEgCIR3672
16.3
mCnCIRB5
20.4
mEgCIR2433
34.3
mEgCIR2525
66.4
mEgCIR2621
86.0
mEgCIR0827
101.0
mEgCIR0906
120.3
mEgCIR0465
136.3
mEgCIR3825
157.7
mEgCIR3538
170.2
mEgCIR0790
183.5
mEgCIR3311
188.9
mEgCIR3407
0.0
mEgCIR3739
24.9
mEgCIR3362
mEgCIR3587
55.6
mEgCIR3293B
69.1
mEgCIR3400
77.9
mEgCIR2110
87.2
mEgCIR3653
102.9
mEgCIR3766
124.9
mEgCIR1977
140.2
mEgCIR3382
145.8
LG12_CHR5
LG13_CHR12
LG14_CHR11
LG15_CHR10
LG16_CHR15
mEgCIR1492
0.0
mEgCIR0037
1.2
mEgCIR0781
5.5
mEgCIR2409
18.8
mEgCIR0177
22.6
mEgCIR3346
23.0
mEgCIR0409
23.6
mEgCIR2670
24.9
mEgCIR3727
35.6
mEgCIR3593
47.0
mEgCIR2590
66.4
mEgCIR2860
70.8
mEgCIR3534
mEgCIR3655
72.2
mEgCIR2144
81.8
mEgCIR3718
86.0
mEgCIR3295
101.7
mEgCIR2380
110.9
mEgCIR0773
111.5
mEgCIR0230
114.6
mEgCIR3292
124.0
mEgCIR3433
125.6
mEgCIR3402
126.4
mEgCIR2569
0.0
mEgCIR3555
5.4
mEgCIR0832
26.5
mCnCIR0038
34.3
mEgCIR2212
38.8
mEgCIR3810
45.0
mEgCIR3399
69.6
mEgEST0259
82.3
mEgCIR3569
0.0
mEgCIR0779
mEgCIR3350
7.3
mEgCIR2427
12.0
mCnCIR0124
21.7
mEgCIR3546
37.7
mEgCIR3607
70.3
mEgCIR0772
80.3
mEgCIR0588
88.1
mEgCIR3633
98.5
mCnCIRg6
0.0
mEgCIR0825
14.5
mEgCIR0243
28.1
mCnCIRE10
37.5
mEgCIR2492
48.1
mEgCIR3213
49.2
mEgCIR2628
54.2
mEgCIR3519
57.3
mEgCIR0146
74.5
mEgCIR2020
83.6
mEgCIR0433
91.7
mEgCIR0366
107.8
mEgCIR3321
114.6
mEgCIR0446
119.8
mEgCIR0445
126.1
mEgCIR3785
131.0
mEgCIR3696
137.8
mEgCIR3639
0.0
mEgCIR3745
15.7
mEgEST0180
20.8
mEgCIR0353
29.2
mEgCIR3298
30.5
mEgCIR2436
32.0
mEgCIR3750
46.9
mEgCIR0782
57.7
Figure S3. Eg9PP oil palm multiparental population consensus genetic map. The map was obtained on the basis of recombination information from the Eg9PP mapping population (n=604). Linkage groups (LG) are numbered according to Billotte et al. (2005, 2010), with corresponding chromosome numbers (CHR) according to Singh et al. (2013). The map distance is given on the left in centimorgans (cM), with marker names on the right.

## Slide 4
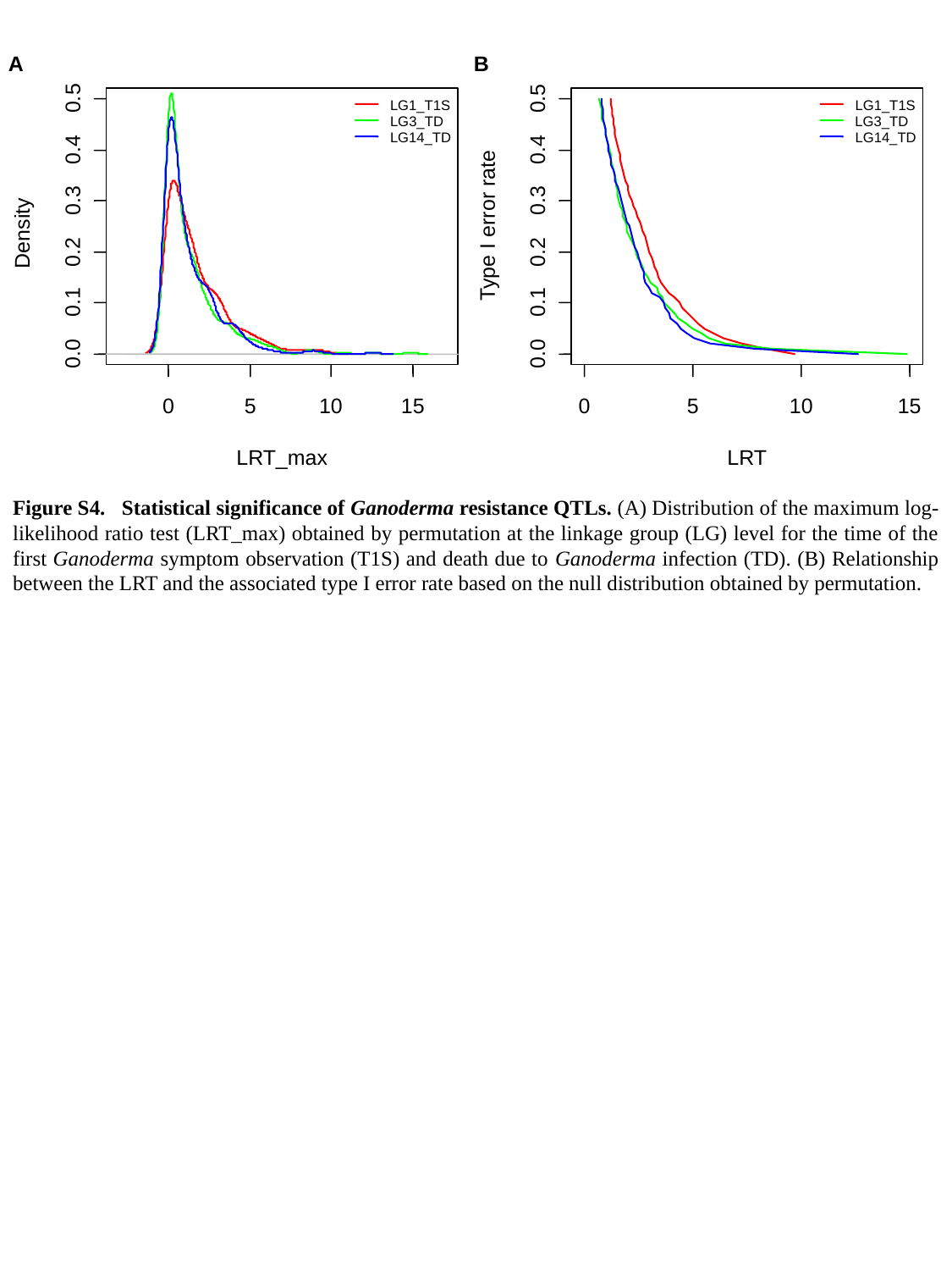

A
B
0.5
0.5
LG1_T1S
LG1_T1S
LG3_TD
LG3_TD
LG14_TD
LG14_TD
0.4
0.4
0.3
0.3
Type I error rate
Density
0.2
0.2
0.1
0.1
0.0
0.0
0
5
10
15
0
5
10
15
LRT_max
LRT
Figure S4. Statistical significance of Ganoderma resistance QTLs. (A) Distribution of the maximum log-likelihood ratio test (LRT_max) obtained by permutation at the linkage group (LG) level for the time of the first Ganoderma symptom observation (T1S) and death due to Ganoderma infection (TD). (B) Relationship between the LRT and the associated type I error rate based on the null distribution obtained by permutation.

## Slide 5
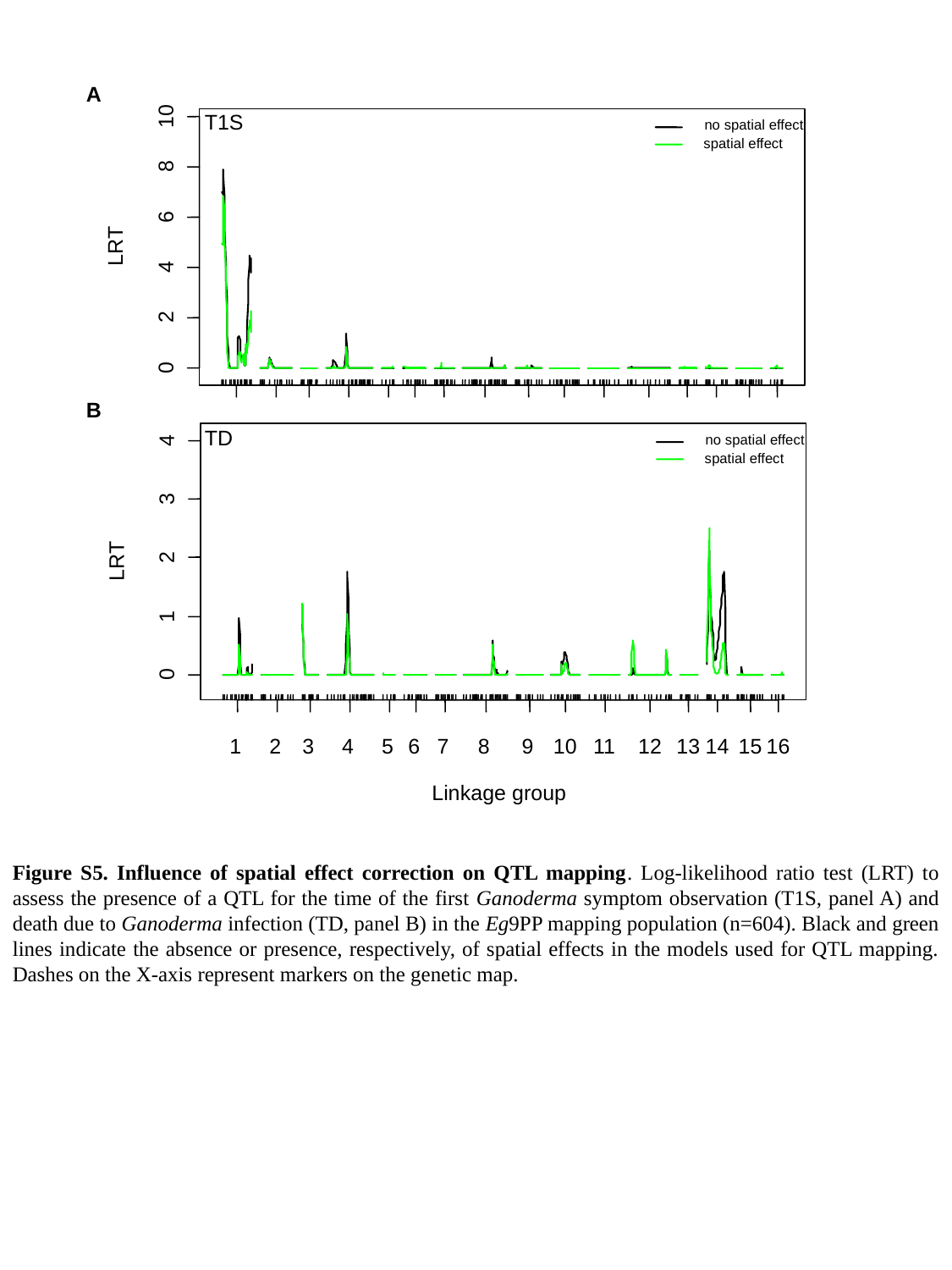

A
10
T1S
no spatial effect
spatial effect
8
6
LRT
4
2
0
B
TD
4
no spatial effect
spatial effect
3
2
LRT
1
0
1
2
3
4
5
6
7
8
9
10
11
12
13
14
15
16
Linkage group
Figure S5. Influence of spatial effect correction on QTL mapping. Log-likelihood ratio test (LRT) to assess the presence of a QTL for the time of the first Ganoderma symptom observation (T1S, panel A) and death due to Ganoderma infection (TD, panel B) in the Eg9PP mapping population (n=604). Black and green lines indicate the absence or presence, respectively, of spatial effects in the models used for QTL mapping. Dashes on the X-axis represent markers on the genetic map.

## Slide 6
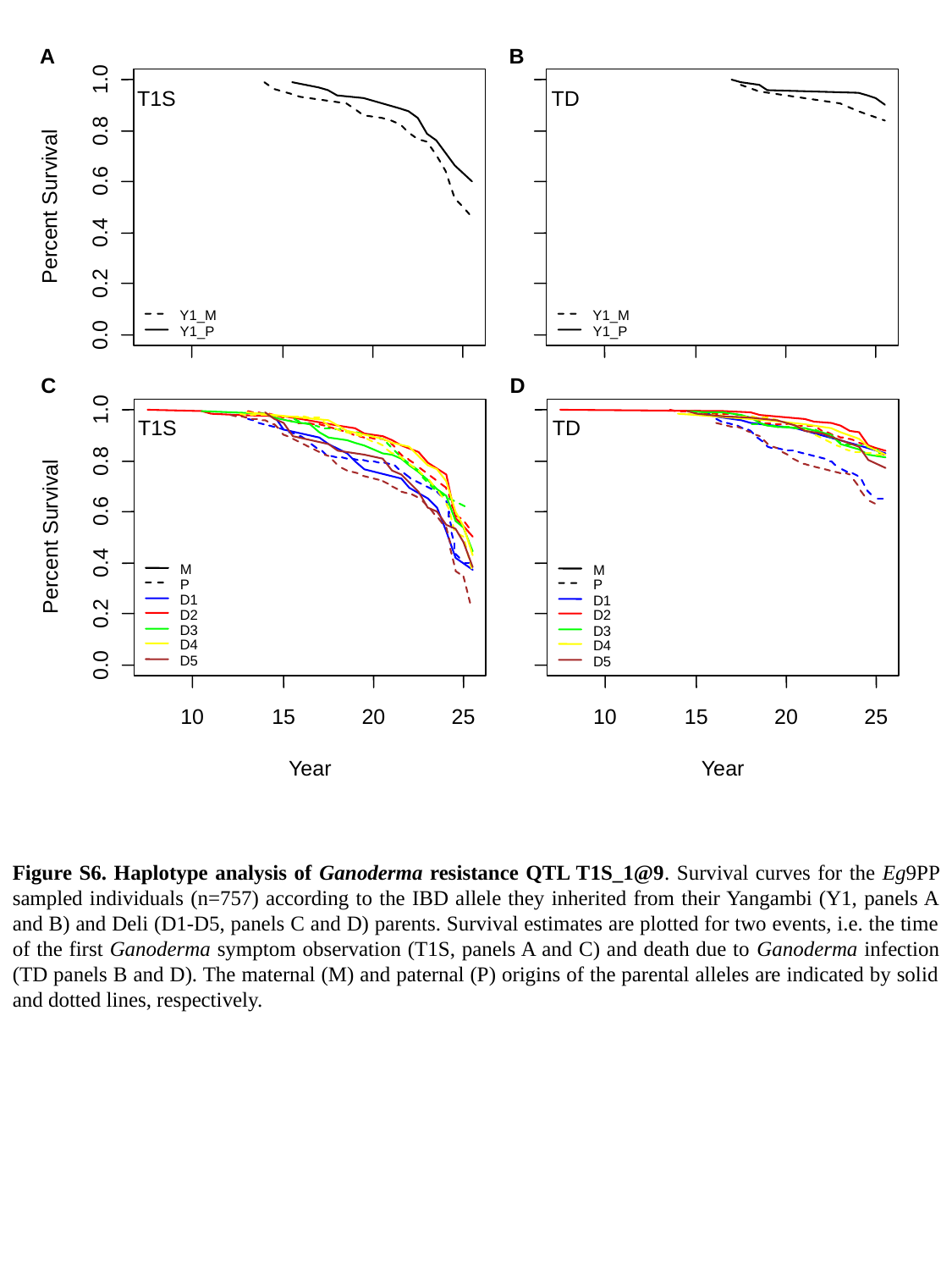

A
B
1.0
0.8
0.6
Percent Survival
0.4
0.2
Y1_M
0.0
Y1_P
Y1_M
Y1_P
T1S
TD
C
D
1.0
0.8
0.6
Percent Survival
0.4
M
P
D1
0.2
D2
D3
D4
D5
0.0
10
15
20
25
Year
M
P
D1
D2
D3
D4
D5
10
15
20
25
Year
T1S
TD
Figure S6. Haplotype analysis of Ganoderma resistance QTL T1S_1@9. Survival curves for the Eg9PP sampled individuals (n=757) according to the IBD allele they inherited from their Yangambi (Y1, panels A and B) and Deli (D1-D5, panels C and D) parents. Survival estimates are plotted for two events, i.e. the time of the first Ganoderma symptom observation (T1S, panels A and C) and death due to Ganoderma infection (TD panels B and D). The maternal (M) and paternal (P) origins of the parental alleles are indicated by solid and dotted lines, respectively.
