# Supplementary material for: Identification of Ganoderma Disease Resistance Loci Using Natural Field Infection of an Oil Palm Multiparental Population
Source: G3 (Bethesda). 2017 Jun 5;7(6):1683–92. doi: 10.1534/g3.117.041764 (PMC5473749; doi:10.1534/g3.117.041764)
Supplement: Supplementary file 7 [file 1683File007.docx]

**Table S2. Six-level *Ganoderma* symptom observation scale on oil palm trees**

| SCORE | OBSERVATION |
| --- | --- |
| 0 | healthy tree, no symptoms |
| 1 | no fruiting bodies, yellow fronds and/or unopened spears |
| 2a or 2b | no fruiting bodies, no foliar symptoms, holes or cracks in the trunk |
| 3a or 3b | fruiting bodies, no foliar symptoms, no trunk symptoms |
| 4a or 4b | fruiting bodies combined with foliar and/or trunk symptoms |
| 5a or 5b | fruiting bodies, standing dead tree |
| 6a or 6b | fruiting bodies, dead and fallen tree |
| 7 | dead tree for unknown reason or absent at the time of the visit |

a: upper stem rot, b: basal stem rot

**Table S7. *Ganoderma* observations in the *Eg*9PP oil palm multiparental population at trial uprooting and risk estimates associated with the first *Ganoderma* symptom observation and death due to *Ganoderma* infection.**

|  | Ganoderma symptom | | | | Death | | | |
| --- | --- | --- | --- | --- | --- | --- | --- | --- |
|  | Observed (%) | Risk^a^ | CI^b^ | p | Observed (%) | Risk | CI | p |
| Eg9PP | 58.5 |  |  |  | 30.50 |  |  |  |
| *Family* |  |  |  |  |  |  |  |  |
| D1Y1 | 52 | 0.48 | 0.32-0.72 | 0.00 | 26.70 | 0.32 | 0.19-0.56 | 0.00 |
| D1L3 | 74.7 | - | - | - | 54.70 | - | - | - |
| D2Y1 | 56 | 0.50 | 0.33-0.75 | 0.00 | 14.70 | 0.20 | 0.10-0.40 | 0.00 |
| D2L1 | 45.2 | 0.42 | 0.29-0.60 | 0.00 | 22.20 | 0.32 | 0.20-0.52 | 0.00 |
| D2L2 | 37.3 | 0.32 | 0.21-0.51 | 0.00 | 17.30 | 0.27 | 0.14-0.50 | 0.00 |
| D2L3 | 69.3 | 0.69 | 0.48-1.01 | 0.06 | 28.00 | 0.41 | 0.24-0.70 | 0.00 |
| D3Y1 | 56.7 | 0.57 | 0.40-0.80 | 0.00 | 33.30 | 0.44 | 0.29-0.67 | 0.00 |
| D3L1 | 65.3 | 0.74 | 0.51-1.09 | 0.13 | 28.00 | 0.46 | 0.27-0.78 | 0.00 |
| D3L2 | 65.3 | 0.71 | 0.49-1.05 | 0.09 | 38.70 | 0.62 | 0.38-1.00 | 0.05 |
| D3L3 | 55.6 | 0.58 | 0.40-0.85 | 0.01 | 40.00 | 0.65 | 0.41-1.02 | 0.06 |
| D4L1 | 53.3 | 0.52 | 0.35-0.79 | 0.00 | 17.30 | 0.27 | 0.14-0.50 | 0.00 |
| D4L3 | 70.7 | 0.69 | 0.47-1.01 | 0.05 | 32.00 | 0.46 | 0.28-0.76 | 0.00 |
| D5L2 | 65.3 | 0.72 | 0.49-1.06 | 0.10 | 32.00 | 0.55 | 0.33-0.92 | 0.02 |
| D5L3 | 65.3 | 0.98 | 0.66-1.43 | 0.90 | 44.00 | 0.96 | 0.61-1.53 | 0.87 |

^a^risk calculated as $exp (\beta)$ using Model 2 (Integrated Log-likelihood = -4638.708 (p=4.72e^-11^, AIC=51.02, BIC=-21.84), $\sigma_{u}^{2}$= 6.19e^-01^, $\sigma_{a}^{2}$= 6.55e^-05^, see Materials and Methods section).

^b^0.95 confidence interval
