# Supplementary material for: Identification of Ganoderma Disease Resistance Loci Using Natural Field Infection of an Oil Palm Multiparental Population
Source: G3 (Bethesda). 2017 Jun 5;7(6):1683–92. doi: 10.1534/g3.117.041764 (PMC5473749; doi:10.1534/g3.117.041764)
Supplement: Supplementary file 8 [file 1683File008.docx]

**Molecular and evolutionary charcterization of the introgressed PgiC locus in sheep's fescue (*Festuca ovina L*.)**

Lena Ghatnekar, Maarit Jaarola, and Bengt Olle Bengtsson

**Genetic material**

The oil palm multi-parent population *Eg*9PP was planted in 1986 in Medan, Indonesia. The whole genetic trial was uprooted in 2012 for replantation. Oil palm genetic material is not maintained as inbred lines because of strong inbreeding depression. However, *Eg*9PP parents are the major funders of Cirad/PalmElit breeding program; hence families belonging to *Eg*9PP can be reproduced by crossing individuals derived from the self-fertilized families of *Eg*9PP parents. Demands for such genetic material should be addressed to PalmElit ([www.palmelit.com](http://www.palmelit.com)).

**Raw Genotype File**

**Table S5. Genotyping of the oil palm multi-parent population *Eg*9PP.**

Genotypes of the 604 individuals of the Eg9PP mapping population and their parents (ID) are presented for the 247 simple sequence repeat markers used in the study. Both unphased parental alleles are given in the two columns below the name of the corresponding marker. The value “0” indicates missing data.

**Marker information file**

**Table S4. Genotyping design and genetic map of the oil palm multi-parent population *Eg*9PP.**

Marker names (Marker) are followed by the primer sequences (Forward and Reverse), information on the genetic map, with linkage group (LG) and position in centiMorgan (cM), information on the physical map (Singh *et al.* 2013), with chromosome (Chr) and physical position (Mb), and their genotyping depending on the family.

**Raw Phenotype file**

**Table S3. Oil palm multi-parent population *Eg*9PP phenotypic data associated to *Ganoderma* infection.**

Occurrence of the events “first Ganoderma symptom observation” (EVENT_T1S) and “death due to *Ganoderma* infection*”* (EVENT_TD) are indicated with the times associated (Y_T1S and Y_TD respectively) for the 1200 *Eg*9PP individuals (ID). Spatial information is indicated with parcel, plot and the geographical coordinates (X_POSITION and Y_POSITION).

**Descriptions of phenotypes**

The infection status by *Ganoderma* *boninense* was recorded biannually on the 1200 *Eg*9PP individuals during 25 years, from the first year after planting (June 1987) to the uprooting of the genetic trial (June 2012). Severity of symptoms was scored blindly on the basis of a six level scale, from 0 for a healthy palm tree to 6 for a dead and fallen palm tree with presence of *Ganoderma* fruiting bodies on the palm trunk (see Table S2 and Fig. S2). A seventh level was defined to record palm tree absent at the visit time or dead for other reason than *Ganoderma* disease. Raw data were then curated and the occurrences of two events were recorded with the time associated, the first *Ganoderma* symptom appearance (T1S, first observation of score 2 to 6) and the death of the palm tree due to *Ganoderma* disease (TD, first observation of score 6).

**Results file**

**Table S6. Results of genome scan for *Ganoderma* infection related traits in the oil palm multi-parent population *Eg*9PP.**

Log-likelihood ratio tests (LRT) are presented for the first Ganoderma symptom observation (T1S) and the death due to *Ganoderma* infection (TD) for the 1006 genetic positions (MatrixID) with corresponding positions on the genetic map. LRT calculated based on the model without inclusion of spatial effect are indicated (_noSPA).

**Table S8. Colocalization of *Ganoderma* resistance QTL and predicted oil palm R-genes**

Markers with known physical position (black) are listed with their corresponding Log-likelihood ratio test for the first Ganoderma symptom observation (LRT_T1S) and the death due to *Ganoderma* infection (LRT_TD), with predicted R-genes (red) with their description (seq_desc).

**Software**

All R scripts, data file and documentation are available at <https://github.com/DenisMarie/Eg9PP_Ganoderma>.
